# Hepatic Deficiency of COP9 Signalosome Subunit 8 Induces Ubiquitin-Proteasome System Impairment and Bim-Mediated Apoptosis in Murine Livers

**DOI:** 10.1371/journal.pone.0067793

**Published:** 2013-07-01

**Authors:** Daoxiong Lei, Faqian Li, Huabo Su, Jinbao Liu, Ning Wei, Xuejun Wang

**Affiliations:** 1 Division of Basic Biomedical Sciences, The University of South Dakota Sanford School of Medicine, Vermillion, South Dakota, United States of America; 2 Department of Hepatobiliary Surgery, Tianjin 4th Central Hospital and The 4th Central Clinical College of Tianjin Medical University, Tianjin, China; 3 Department of Pathology and Laboratory Medicine, University of Rochester Medical Center, Rochester, New York, United States of America; 4 Vascular Biology Center and Department of Pharmacology and Toxicology, Medical College of Georgia, Georgia Regents University, Augusta, Georgia, United States of America; 5 Department of Pathophysiology, Guangzhou Medical College, Guangzhou, Guangdong, China; 6 Department of Molecular, Cellular and Developmental Biology, Yale University, New Haven, Connecticut, United States of America; Loyola University Chicago, United States of America

## Abstract

The COP9 signalosome (CSN), an evolutionally highly conserved protein complex composed of 8 unique subunits (CSN1 through CSN8) in higher eukaryotes, is purported to modulate protein degradation mediated by the ubiquitin-proteasome system (UPS) but this has not been demonstrated in a critical mitotic parenchymal organ of vertebrates. Hepatocyte-specific knockout of the Cops8 gene (HS-Csn8KO) was shown to cause massive hepatocyte apoptosis and liver malfunction but the underlying mechanism remains unclear. Here, we report that Csn8/CSN exerts profound impacts on hepatic UPS function and is critical to the stability of the pro-apoptotic protein Bim. Significant decreases in CIS (cytokine-inducible Src homology 2 domain-containing protein), a Bim receptor of a cullin2-based ubiquitin ligase, were found to co-exist with a marked increase of Bim proteins. Csn8 deficiency also significantly decreased 19S proteasome subunit Rpt5 and markedly increased high molecular weight neddylated and ubiquitinated proteins. The use of a surrogate UPS substrate further reveals severe impairment of UPS-mediated proteolysis in HS-Csn8KO livers. Inclusion body-like materials were accumulated in Csn8 deficient hepatocytes. In addition to Bim, massive hepatocyte apoptosis in HS-Csn8KO livers is also associated with elevated expression of other members of the Bcl2 family, including pro-apoptotic Bax as well as anti-apoptotic Bcl2 and Bcl-XL. Increased interaction between Bcl2 and Bim, but not between Bcl2 and Bax, was detected. Hence, it is concluded that hepatic CSN8 deficiency impairs the UPS in the liver and the resultant Bim upregulation likely plays an important role in triggering hepatocyte apoptosis via sequestering Bcl2 away from Bax.

## Introduction

The ubiquitin-proteasome system (UPS) constitutes a major degradation pathway for intracellular proteins and plays important roles in virtually all cellular processes. UPS-mediated proteolysis involves two essential steps: ubiquitination and proteasomal degradation. Ubiquitination tags the target proteins with a chain of the small protein molecule ubiquitin and the ubiquitin chain serves as a signal of the target proteins for degradation by the 26S proteasome [1]. The 26S proteasome is a multi-unit protein proteolytic machine composed of a cylinder-shaped core particle (i.e., the 20S proteasome) and the 19S regulatory particle (i.e., the 19S proteasome) at one or both ends of the 20S. Proteasomal proteolytic enzymes reside, and peptide cleavage takes place, in the 20S proteasome. The 19S serves to regulate protein degradation by the 20S proteasome. The 19S proteasome consists of two distinct substructures known as the “lid” and the “base”. The base of the 19S directly interacts with the α (outer) ring of the 20S, responsible for unfolding the target protein and regulating the gating of the 20S. The lid plays a critical role in recognizing and binding polyubiquitinated target proteins and the removal of ubiquitin from the target proteins for ubiquitin recycling [2]. Besides the 19S, the 11S proteasome which is formed by heteropolymerization of PA28α and PA28β or by homopolymerization of PA28γ, can also associate with the 20S to form hybrid proteasomes (11S-20S-19S) or 11S-associated 20S proteasome complexes [3]. Upregulation of 11S proteasomes by PA28α overexpression can facilitate the degradation of misfolded proteins by the UPS [4,5]. UPS involvement in the pathogenesis of many forms of liver disease is well documented [6,7]. Accordingly, the UPS has been suggested as a therapeutic target for many liver diseases [8,9]. Hence, it is important to achieve a better understanding on the regulation of the UPS and the consequence of UPS dysfunction in the liver.

The COP9 signalosome (CSN) consists of 8 unique protein subunits (CSN1 through CSN8) in mammalian cells. It is an evolutionarily conserved multifunctional protein complex that is essential in plants and animals [10]. The CSN has an intrinsic metalloproteinase that removes the ubiquitin-like protein Nedd8 from cullins via a process known as deneddylation [11,12]. CSN-mediated deneddylation is purported to regulate the assembly and catalytic dynamics of various cullin-RING ubiquitin ligases (CRLs) [13–15], a large family of ubiquitin E3’s that control the ubiquitination and degradation of many proteins. CSN subunit 8 (CSN8) is the smallest and the least conserved subunit of the CSN complex. Conditional knockout of the *csn8* gene in mice has helped determine the essential role of Csn8/CSN in antigen-induced initiation of T cell proliferation [16] and in the postnatal cardiac development and heart function [17,18]. Csn8 deficiency was shown to cause UPS impairment in the heart but this has not been examined in other organs [17].

We have previously reported that Csn8/CSN is essential to hepatocyte survival and effective proliferation in mice [19]. In livers deficient of Csn8, hepatocytes showed massive apoptosis, resulting in marked proliferation of the oval cells and cells of the biliary lineage, and extensive interstitial fibrosis. The liver pathology induced by hepatocyte-specific Csn8 knockout (HS-Csn8KO) recapitulates the sequalea of chronic hepatic injury such as viral hepatitis [19]. Hence, dissecting the molecular links between Csn8 deficiency and the resultant hepatocyte apoptosis will shine light on the pathogenesis of a large group of liver diseases.

Hepatocyte apoptosis is a common mechanism of liver injury including hepatitis, liver fibrosis and cirrhosis. It appears that hepatocyte apoptosis can be induced via both the extrinsic and the intrinsic pathways [20]. In the intrinsic pathway, mitochondria release several apoptosis-promoting factors in response to diverse death signals via sensor molecules, such as BH3-only proteins. Activation of pro-apoptotic proteins, such as Bax, can trigger a series of events that lead to the activation of caspases [21], resulting in DNA fragmentation and cellular morphologic changes characteristic of apoptosis. Notably, the Bcl2 family proteins constitute a critical intracellular checkpoint in the mitochondrial pathway of apoptosis [22]. The Bcl2 family consists of both anti-apoptotic and pro-apoptotic proteins. The anti-apoptotic members, including Bcl2, Bcl-xL and Mcl1, display sequence conservation through four Bcl2 homology domains (BH1 through BH4), whereas the pro-apoptotic members are composed of more fully conserved ‘multidomain’ proteins (e.g., Bax and Bak) possessing homology in BH1~BH3, as well as the BH3-only proteins (e.g., Bid, Bim, Bad) which harbor only the BH3 domain [22,23]. The BH3-only proteins can be further categorized into the ‘activator’ (e.g., Bim, tBid) and the “sensitizer” (e.g., Bad, Noxa) subgroups [24,25]. In viable cells, the anti-apoptotic proteins, Bcl2 and Bcl-xL, bind to and repress the multidomain pro-apoptotic proteins Bax and Bak, and keep them in inactive monomers. In response to cellular stresses, BH3-only proteins Bim and tBid can bind to Bcl2 or Bcl-xL, thereby neutralize their anti-apoptotic effects, and relieve Bax or Bak from Bcl2-bound complexes [23]; or alternatively Bim and tBid directly activate Bax and Bak [25,26], thereby initiating caspase activation and cell death. Therefore, the interaction and the functional balance between pro-apoptotic and anti-apoptotic Bcl2 family proteins in a cell determine the fate of the cell [21].

Using a *Cre-loxP*-mediated HS-Csn8KO mouse model, we have uncovered in the present study that Csn8 deficiency leads to UPS derangement and functional impairment, which are associated with massive apoptosis. We also presented the first evidence that the upregulation of pro-apoptotic protein Bim and its interaction with Bcl2 may have mediated the Bax-dependent activation of the intrinsic pathway of hepatocyte apoptosis in Csn8 deficient livers.

## Materials and Methods

### 1. Animal models

HS-Csn8KO mice were generated with the use of the *Cre-loxP* system as previously described [19]. Briefly, Csn8^flox/flox^ mice were cross-bred with the *Cre* transgenic mice (Alb-Cre) in which the *Cre* expression is controlled by an albumin promoter [27]. Ultimately, littermate mice with a genotype of either Csn8^flox /flox^::Alb-Cre(+) or Csn8^flox /flox^::Alb-Cre(-) were used in the present study as the HS-Csn8KO or the control (CTL) mice, respectively.

The generation and characterization of transgenic mice with ubiquitous overexpression of a surrogate UPS substrate GFPdgn were previously described [28]. GFPdgn is an enhanced green fluorescence protein (GFP) modified by carboxyl fusion of degron CL1 [28]. Using a cross-breeding strategy similar to what we previously reported [17], the GFPdgn transgene was introduced into the HS-Csn8KO and CTL background via cross-breeding, yielding the HR-Csn8KO-GFPdgn group and the CTL-GFPdgn group used in the present study.

The care and use of animals in this study was approved by the Institutional Animal Care and Use Committee (IACUC) of the University of South Dakota.

### 2. Protein extraction, co-immunoprecipitation and western blot analysis

Liver tissue samples were homogenized in sample buffer with phosphatase inhibitor cocktail (Sigma, Saint Louis, MO) and protease inhibitor cocktails (Complete; Roche, IN). The protein concentration was determined with BCA reagents (Pierce, Rockford, IL). Equal amounts of proteins were electrophoresed on 10%, 12% or 15% SDS-PAGE and transferred to PVDF membranes. Western blots were performed with following antibodies: Rpn2, Rpn8, Rpn10, Rpt5, PA28α, PA28β, PA28γ, VHL (von Hippel–Lindau), HIF1α (hypoxia inducible factor 1α), and Csn8 (Biomol), Bim and Bcl-xL (Chemicon), Bax and Bcl2 (BD Pharmingen Tech.), Nedd8 (Alexis), β-tubulin and ubiquitin (Sigma), CIS (cytokine-inducible Src homology 2 domain-containing protein) and GFP (Santa Cruz Biotechnology), and 20S subunit β5 (custom made). Horseradish peroxidase–conjugated anti-mouse, -rabbit, or -rat secondary antibodies (Santa Cruz) were used to probe the bound primary antibodies and detected using ECL advanced detection reagents (Amersham Pharmacia Biotech, Piscataway, NJ). A VersaDoc Model 3000 Imaging System (Bio-Rad Laboratories, Hercules, CA) was used to visualize and digitalize the western blot images. The densitometry of the western blots was performed with the Quantity-One software (Bio-Rad).

For co-immunoprecipitation, the proteins (400µg) solubilized in RIPA lysis buffer were incubated respectively with primary antibody against Bim, Bcl2, or Bax (2µg/ml) at 4°C for 3 hours and precipitated with protein-A/G–Sepharose (Santa Cruz) at 4°C for 2 hours. After centrifugation, the pellets were washed with RIPA buffer for four times. The immunoprecipitates dissolved in SDS-sample buffer were analyzed by western blotting.

### 3. TUNEL assay

The terminal deoxynucleotidyl transferase-mediated dUTP nick-end labeling (TUNEL) assay was performed using the TUNEL Cell Death Detection Kit (Roche Diagnostics, Mannheim, Germany), as previously described [19]. The label solution only (without terminal transferase) instead of TUNEL reaction mixture was used as negative control. At least 300 nuclei were counted from three different random fields in each liver, and data from 3 livers per group are presented. TUNEL index was expressed as a ratio of the number of TUNEL positive nuclei and the total number of nuclei stained with DAPI.

### 4. Fluorescence microscopy

GFPdgn direct fluorescence of cryosections from paraformaldehyde fixed mouse livers, and the TUNEL or DAPI labeled fluorescence in cryosections were visualized using a Zeiss Axiovert 200M epi-fluorescence microscope (Carl Zeiss MicroImaging GmbH, Germany).

### 5. Immunohistochemistry

Tissue samples were collected from the right lobe of the liver, fixed in 10% formalin, and processed for paraffin-embedding. Serial 5-μm paraffin sections were used for Csn8 immunohistochemistry as previously described [19]. Briefly, paraffin-embedded sections were de-paraffinized and rehydrated. The slides were further incubated in 1N HCl for 8 minutes at 65°C and blocked with 0.3% H_2_O_2_ for 20 minutes, washed in PBS, and blocked with 1% (w/v) bovine serum albumin (BSA) in PBS. The BSA-blocked slides were incubated with rabbit anti-Csn8 antibody for 1 hour at room temperature. Biotinylated anti-rabbit secondary antibody was used at a 1:200 dilution and visualized with peroxidase reaction using the ABC Vectastain kit (Vector Laboratories, Burlingame, CA).

### 6. Electron microscopy

This was performed essentially as previously described [18]. Mice were anesthetized with isoflurane and the tissue samples from the left and right lobes of livers were fixed in 3.5% glutaraldehyde in 100 mM cacodylate buffer (pH 7.3). At least 3 tissue samples from each lobe of the liver were chosen randomly for ultrastructural analysis. Two mice for each genotype were examined. Toluidine (Tol) blue stain of the semi-thin sections of resin-embedded tissue was performed and observed using bright-field light microscope to help orientate the tissue sample and select areas of interest for ultra-thin sectioning. Ultrathin sections were picked up on nickel grids, dried and etched with a saturated solution of sodium m-periodate and 0.1N HCl. Thin sections were counterstained with uranyl acetate and lead citrate. The sections were viewed in a Zeiss, Omega 912 electron microscope at 100 kV.

### 7. Statistical analyses

All quantitative data were presented as mean ± SD unless otherwise indicated and analyzed using Student *t*-test for unpaired two group comparison. A p-value <0.05 is considered statistically significant.

## Results

### 1. Csn8 deficiency increases high molecular weight (HMW) Nedd8 and ubiquitin conjugates and leads to UPS derangement in mouse livers

**Figure 1:**
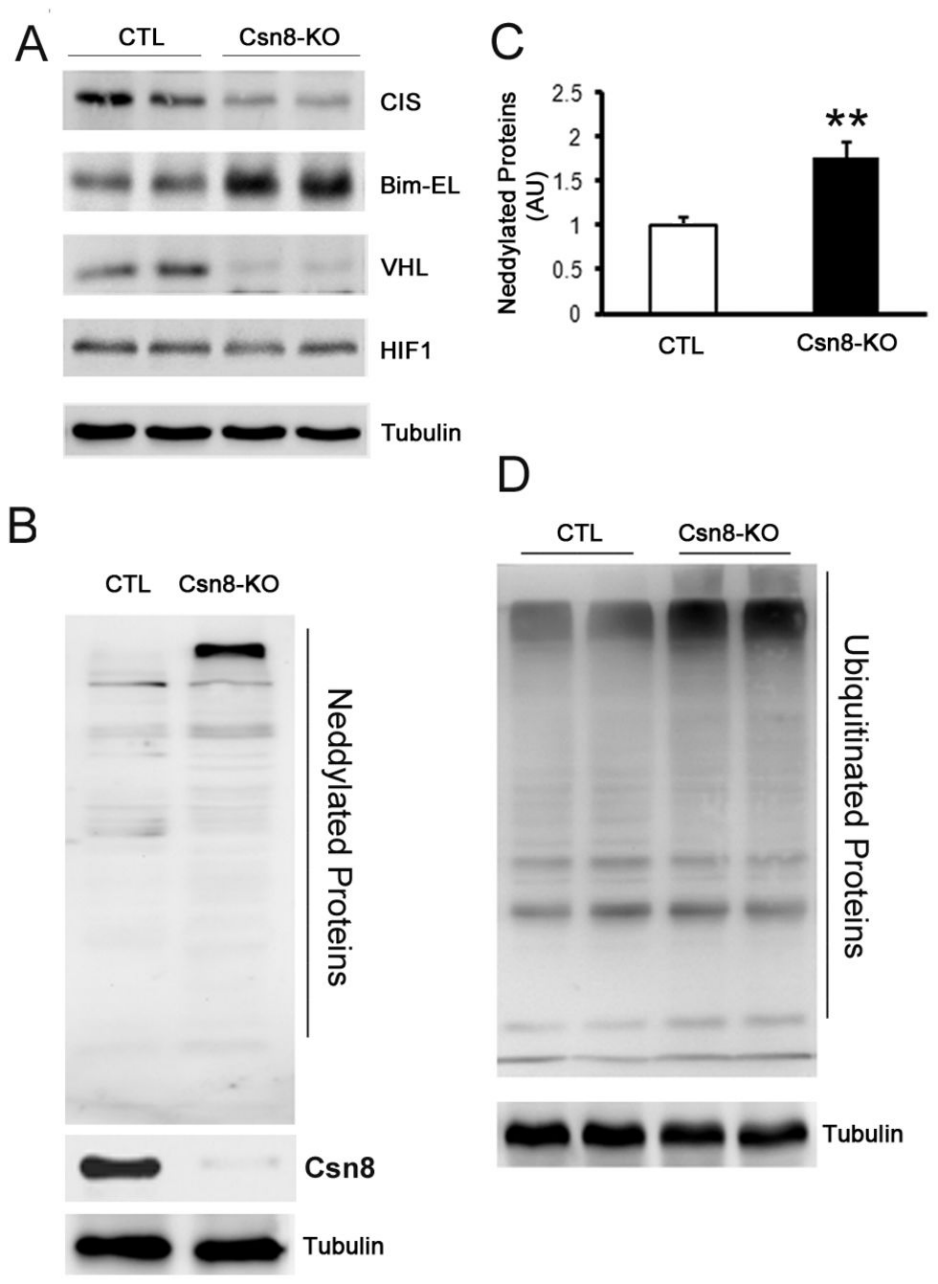
Effects of HS-Csn8KO on deneddylation and CRLs activities in mouse liver at 4 weeks of age. Liver tissue samples were collected from Csn8^flox/flox^::Cre^TG^ (referred to as HS-Csn8KO) and Csn8^flox/flox^::Cre ^NTG^ (referred to as CTL) littermate mice. (**A**) Representative images of western blot analyses for the indicated CRLs and their substrates. (**B**, **C**) Western blot analysis for hepatic total Nedd8 conjugates. Representative images (B) and pooled quantitative data (C) are presented. **p<0.01 vs. CTL. (**D**) Representative images of western blot analysis for hepatic total ubiquitin conjugates. Total ubiquitinated proteins were increased by >50% in HS-Csn8KO livers compared with the CTL (p<0.01). n=4 mice/group, student’s t-test.

To dissect the molecular link between Csn8 deficiency and the activation of apoptotic pathways, we examined changes in the UPS in HS-Csn8KO livers. Significantly increases in the mono-neddylated form of cullins have been reported in HS-Csn8KO livers [19], confirming that CSN-mediated cullin deneddylation in hepatocytes requires Csn8. CSN-mediated cullin deneddylation is purported to prevent self-ubiquitination and destabilization of CRLs. Consistent with this postulate, we detected in HS-Csn8KO livers significant decreases in CIS (cytokine-inducible Src homology 2 domain-containing protein) and VHL (p<0.01, Figure 1A). CIS and VHL serve as the receptor protein for Bim-EL and HIF1α in the Elongin B/C-Cullin2-CIS and the Elongin B/C-Cullin2-VHL ligases that target Bim-EL and HIF1α for degradation, respectively [29,30]. In agreement with the decrease of CIS, the extra-long form of Bim (Bim-EL) protein which is a known substrate of CIS [29], was significantly increased in HS-Csn8KO livers compared with the CTL (p<0.01). By contrast, the protein level of HIF1α, a substrate of VHL [31], showed no discernible increase (p>0.05, Figure 1A).

We previously observed marked increases in the mono-neddylated form of cullins in HS-Csn8KO livers [19]; here we report that HMW nedd8 conjugates were significantly higher in HS-Csn8KO liver than the CTL at 4 weeks of age (p<0.01, Figure 1B, 1C). Interestingly, the abundance of HMW ubiquitin conjugates were also remarkably greater in HS-Csn8KO livers than in the CTL (p<0.01, Figure 1D).

**Figure 2:**
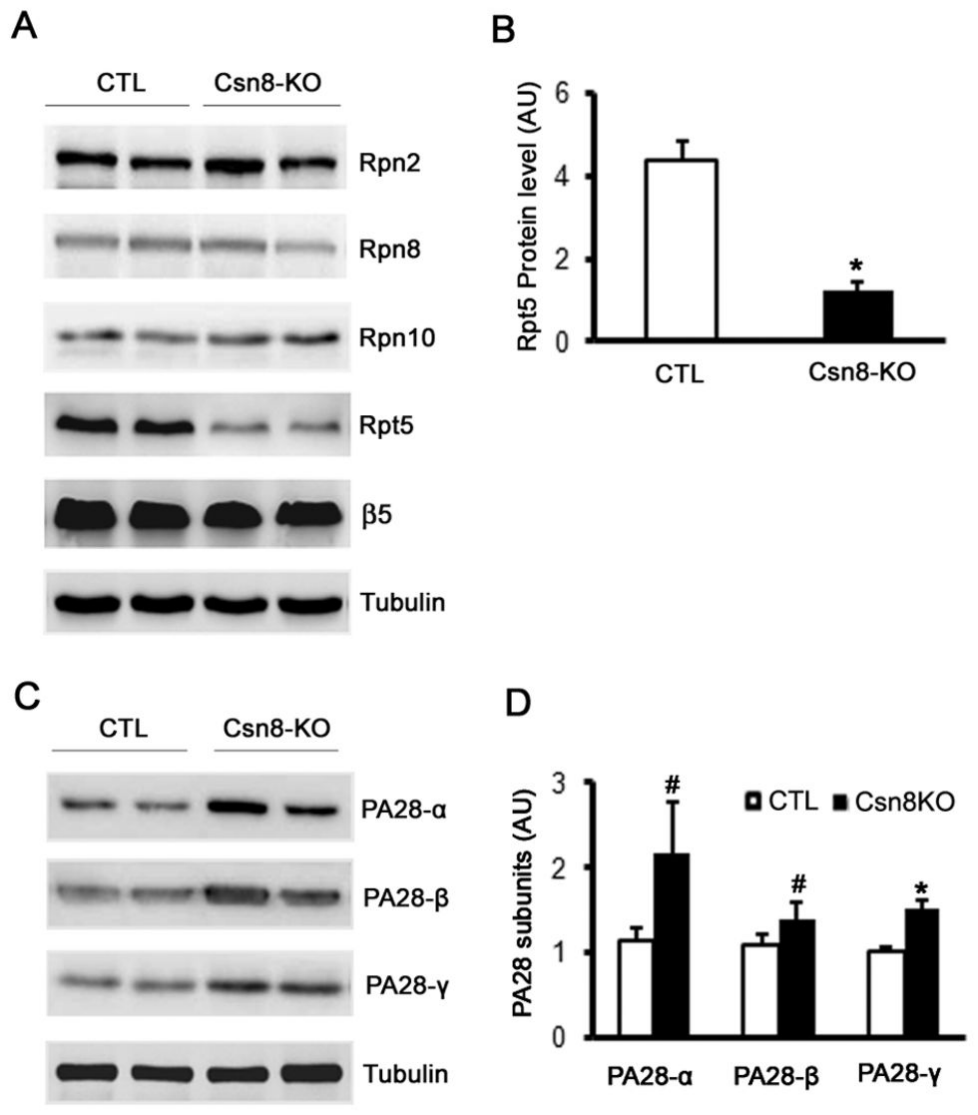
HS-Csn8KO yields differential effects of on proteasome subcomplexes in mouse livers. (**A**) Representative images of western blot analyses of the indicated subunits of the 19 and the 20S proteasome in the liver of 4-week-old mice. (**B**) Pooled densitometry data of the western blot analysis of Rpt5 as illustrated in panel A. (**C**, **D**) HS-Csn8KO resulted in the upregulation of three subunits of the 11S proteasome. β-Tubulin was probed as the loading control. AU denotes arbitrary unit. n=4 mice/group; #p<0.05, *p<0.01 vs. CTL; Student’s *t*-test.

The CSN holo-complex shares morphology and subunit domain structure similarities with the lid of the 19S proteasome; hence, it has been proposed that the CSN may serve as an alternative “lid” for the 19S proteasome to compete with the lid of the 19S proteasome in binding the base of the 19S. Csn8 deficiency is known to reduce the abundance of CSN holo-complex in mice [16,17]. Therefore, we examined the subunit protein levels of the 19S proteasome in HS-Csn8KO mouse livers. Csn8 deficiency show no discernible effect on the protein levels of Rpn2, Rpn8, and Rpn10, three 19S lid subunits. Interestingly, the level of a 19S base subunit Rpt5 was significantly lower in HS-Csn8KO liver than the CTL (p<0.01, Figure 2A, 2B). HS-Csn8KO showed no discernible effect on the protein level of 20S subunit β5. Strikingly, all three subunits (PA28-α, -β, -γ) of the 11S proteasome showed greater protein expression in HS-Csn8KO livers than in the CTL (p<0.05, 0.01; Figure 2C, 2D).

### 2. Csn8 deficiency impairs UPS proteolytic function in the liver

**Figure 3:**
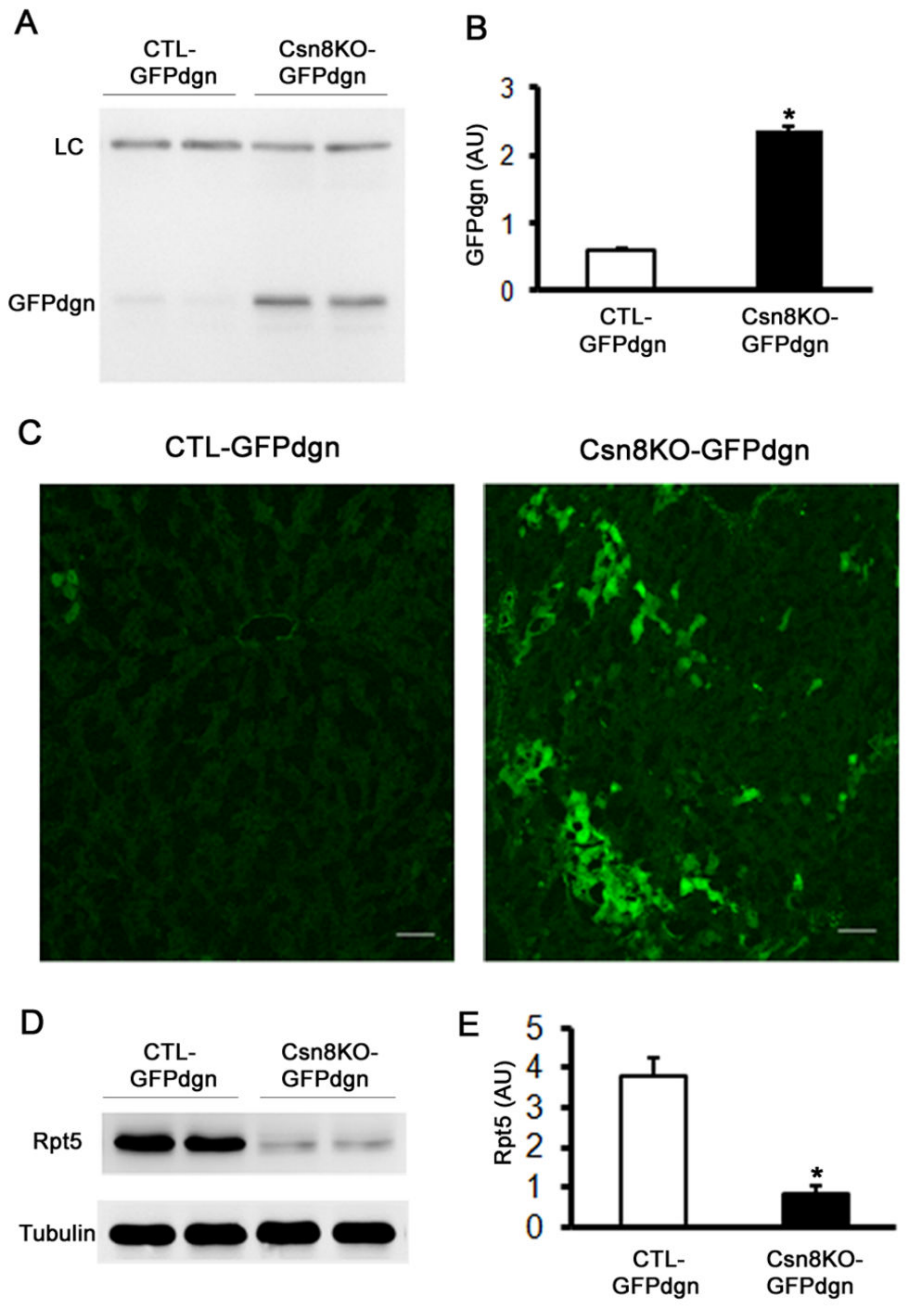
HR-Csn8KO impairs UPS proteolytic function in mouse livers. CTL::GFPdgn tg and H-Csn8KO::GFPdgn tg littermate mice resulting from cross-breeding between GFPdgn tg and HS-Csn8KO mice were used at 4 weeks of age. (**A**, **B**) Representative image (A) and pooled densitometry data (B) of western blot analyses of hepatic GFPdgn. A nonspecific band at a molecular weight of ~80kDa is used as the loading control (LC). (C) Representative fluorescence confocal micrographs of GFPdgn direct fluorescence. Scale bar=50 µm. (D, E) Western blot analyses of Rpt5 in this cohort of mice. *p<0.01 vs. CTL::GFPdgn, n=4 mice/group; Student’s t-test.

Proteolysis via the UPS plays important roles in a variety of basic cellular processes. The CSN may act as a UPS regulator by cullin neddylation of CRLs and potential association with the 19S proteasome [32,33]. To investigate UPS proteolytic function in intact animals, we previously established a transgenic mouse model that ubiquitously overexpresses GFPdgn, a modified green fluorescence protein (GFP) with its carboxyl fusion of degron CL1. GFPdgn, which is similar to GFP^u^ [34], has proven to be a surrogate substrate of the UPS [28]. GFPdgn protein levels inversely correlate to UPS proteolytic function in the cell [35,36]. Therefore, we introduced GFPdgn into the CTL and HS-Csn8KO mice via cross-breeding, to explore the *in vivo* impact of hepatic Csn8 deficiency on UPS-mediated proteolysis in the liver. HS-Csn8KO increased significantly hepatic GFPdgn protein levels (*p*<0.01; Figure 3A, 3B). The accumulation of GFPdgn was further confirmed by the increased intensity of GFP direct fluorescence in HS-Csn8KO mouse livers (Figure 3C). These results compellingly demonstrate that Csn8 deficiency in liver leads to severe impairment of UPS-mediated proteolysis. Meanwhile, the quantitative western blot analyses confirmed a dramatic decline in the protein level of subunit Rpt5 of the 19S proteasome in HS-Csn8KO-GFPdgn livers (Figure 3D, 3E), indicating that UPS malfunction resulted from Csn8 deficiency in the liver is closely associated with the down regulation of a key subunit of the base of 19S proteasomes.

### 3. Morphological characterization of HS-Csn8KO livers

**Figure 4:**
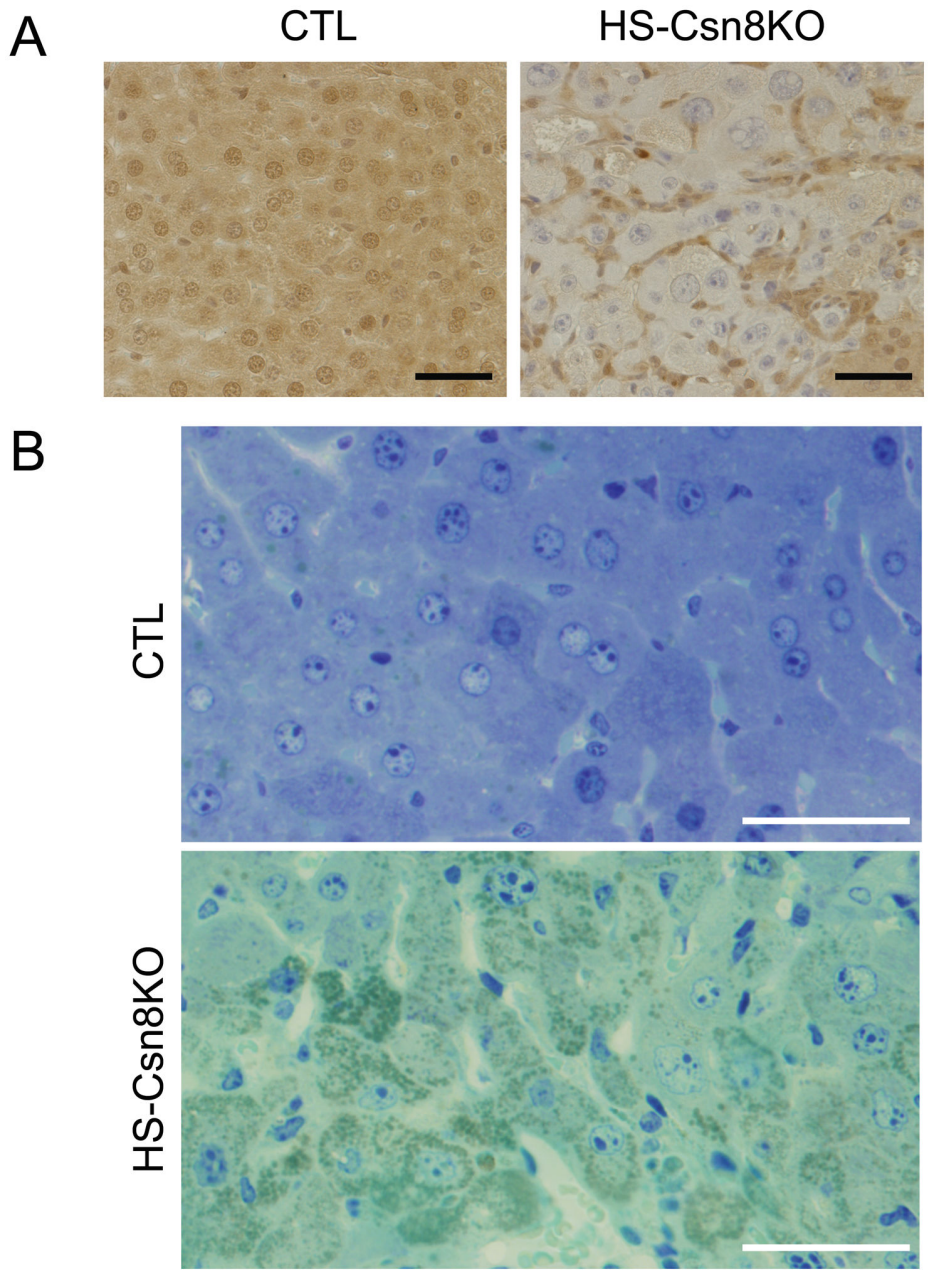
Hepatocytomegaly, nuclear megaly, and increased cytoplasmic inclusions in HS-Csn8KO mouse livers. (**A**) Representative images of Csn8 immunohistochemistry (brown). Liver tissues were sampled from HS-Csn8KO and CTL littermate mice at 6 weeks of age. Immunohistochemistry for Csn8 was performed as described in Methods. The nuclei were counter-stained blue with hematoxylin. (**B**) Representative images of toluidine blue stain of semi-thin sections of resin-embedded liver tissues sampled from HS-Csn8KO and CTL littermate mice at 4 weeks of age. Scale bar=20µm.

Consistent with protein accumulation resulting from impaired UPS function in HS-Csn8KO livers, immunohistochemistry examination revealed that Csn8-negative hepatocytes displayed striking enlargement in cell size and nuclear size, compared with those in the littermate CTL at 6 weeks of age (Figure 4A). Toluidine blue stain of the semi-thin sections of resin-embedded liver tissue samples of 4-week-old mice revealed a large number of greenish granules or puncta in the cytoplasm of hepatocytes of HS-Csn8KO but not CTL livers (Figure 4B).

**Figure 5:**
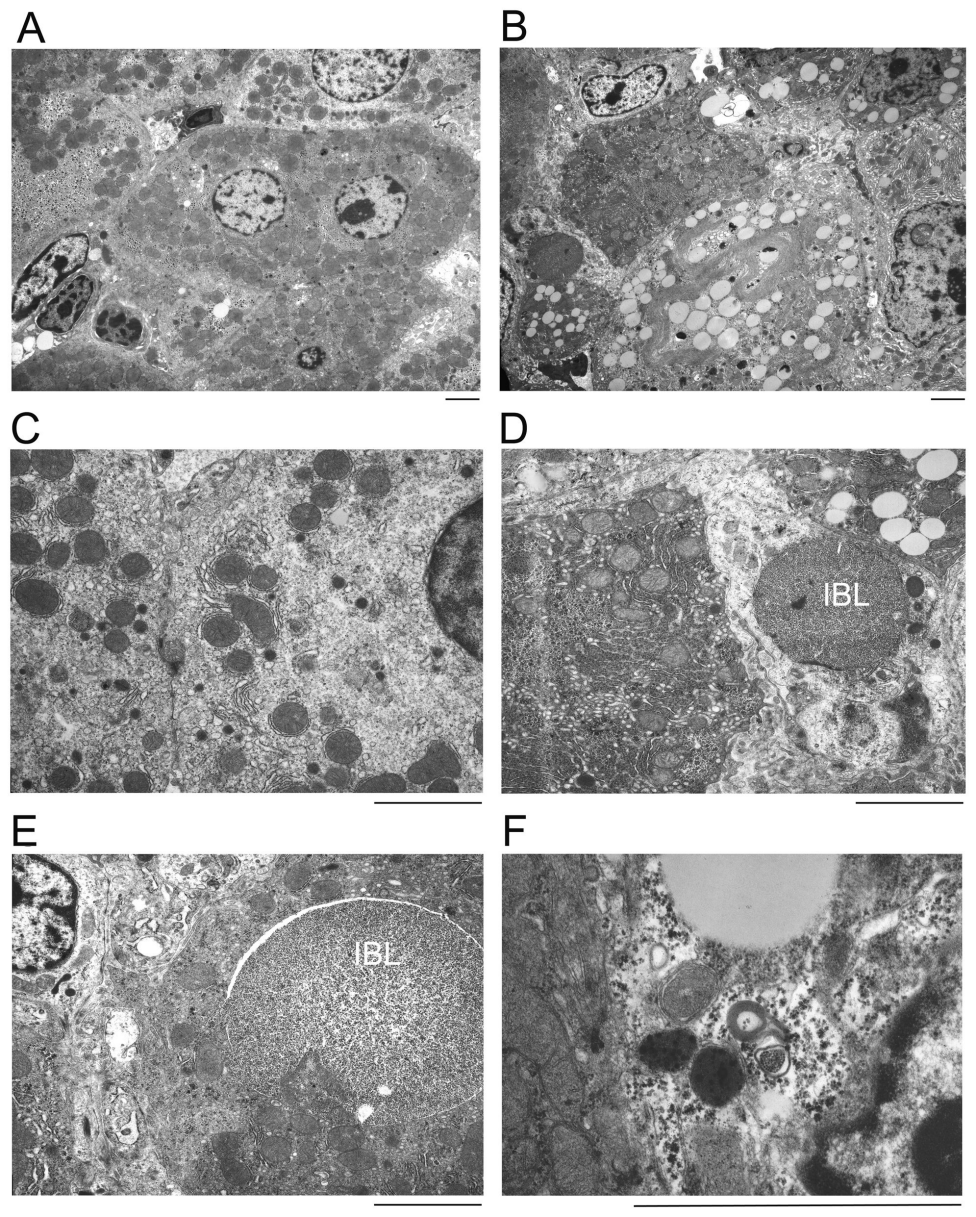
Representative electron micrographs of liver tissue sections. Liver tissues samples from the CTL (A, C) and HS-Csn8KO (B, D ~ F) littermate mice at 4 weeks of age were processed for transmission electron microscopy. IBL, inclusion-body like structure; Scale bar=1µm.

Transmission electron microscopy (TEM) of the liver tissue from 4-week-old CTL mice revealed that hepatocytes were enriched with mitochondria which were frequently associated with rough endoplasmic reticulum. The hepatocyte in CTL livers usually contained a centrally located sphere-shaped nucleus which displayed a smooth outline profile and a diameter of approximately 2µm. Electron-lucent or -dense vacuoles and autophagosomes were rare in the hepatocytes of CTL livers (Figure 5A, 5C). In HS-Csn8KO mice, however, the nucleus of hepatocytes was markedly enlarged and lost its normal spherical shape. The outline of the nuclear envelop became rather rough and cytoplasmic invaginations resulting cytoplasmic inclusions were sometimes seen in the nuclear territory (Figure 5B). In HS-Csn8KO livers, a prominent feature is the presence of a large number of electron-lucent vacuoles of various sizes (diameter ranging from 0.2µm to 0.8µm) in the majority of hepatocytes. Electron dense vacuoles with a diameter of approximately 0.2µm were also more frequently observed in HS-Csn8KO than in CTL hepatocytes. Large inclusion body-like (IBL) structures full of homogenous dense fine granules were present in a subpopulation of hepatocytes in HS-Csn8KO livers. Some of the IBL structures appeared to be membrane-bound (Figure 5D) but others were not (Figure 5E); both often showed a clear boundary from their surroundings. In addition, multi-layered myelin-like structures (Figure 5B) and autophagosome-like structures of various stages (Figure 5F) were also observed in HS-Csn8KO hepatocytes. TEM feature of apoptosis was also observed in HS-Csn8KO livers (*data not shown*).

### 4. Temporal changes in apoptosis in HS-Csn8KO mouse livers

**Figure 6:**
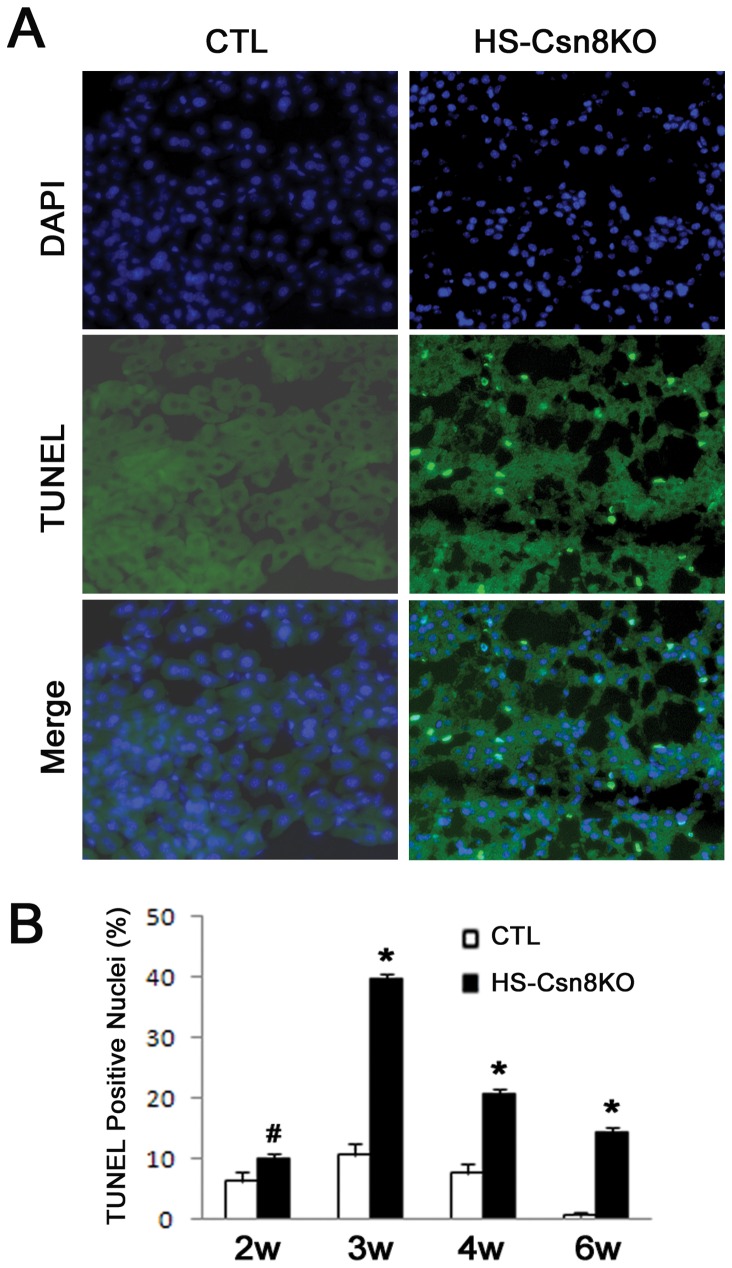
Temporal characterization of hepatocyte apoptosis in HS-Csn8KO mice. (A) Representative images of TUNEL staining of liver cryosections from 6-week-old mice. Scale bar: 50µm. (B) The time course of changes in TUNEL positivity in the liver during postnatal 6 weeks. Four livers each group and 300 nuclei per liver were counted. #p<0.05, *p<0.01 vs. CTL.

We have previously reported massive apoptosis in the liver of HS-Csn8KO mice at 4 weeks of age [19]. Our previous characterization of this HS-Csn8KO mouse model has revealed that the depletion of Csn8 protein starts in between postnatal week 1 and 2 and a significant decrease of Csn8 protein is evidenced at week 2 [19]. Hence, we characterized in the present study the time course of hepatocyte apoptosis in HS-Csn8KO mice during the first 6 weeks after birth. We observed that hepatocyte apoptosis, as revealed TUNEL assays, was significantly increased at week 2, peaked at week 3, and remained at a significantly increased level at 4 and 6 weeks after birth in HS-Csn8KO mice, compared with the littermate CTL (Figure 6), consistent with the time course of Csn8 protein depletion.

Notably, we have previously observed cardiomyocyte massive necrosis in cardiomyocyte-restricted Csn8KO mice produced using the same Csn8-floxed allele [17]; however, the same *in vivo* Evans blue dye permeability assay as we used in cardiac Csn8KO mice [17], showed no discernible increases in hepatocyte membrane permeability in the HS-Csn8KO mice at 4 weeks of age (*data not shown*), indicating that TUNEL positivity in HS-Csn8KO liver results from primarily apoptosis.

### 5. Increased expression of the *Bcl2* family members in HS-Csb8KO livers

**Figure 7:**
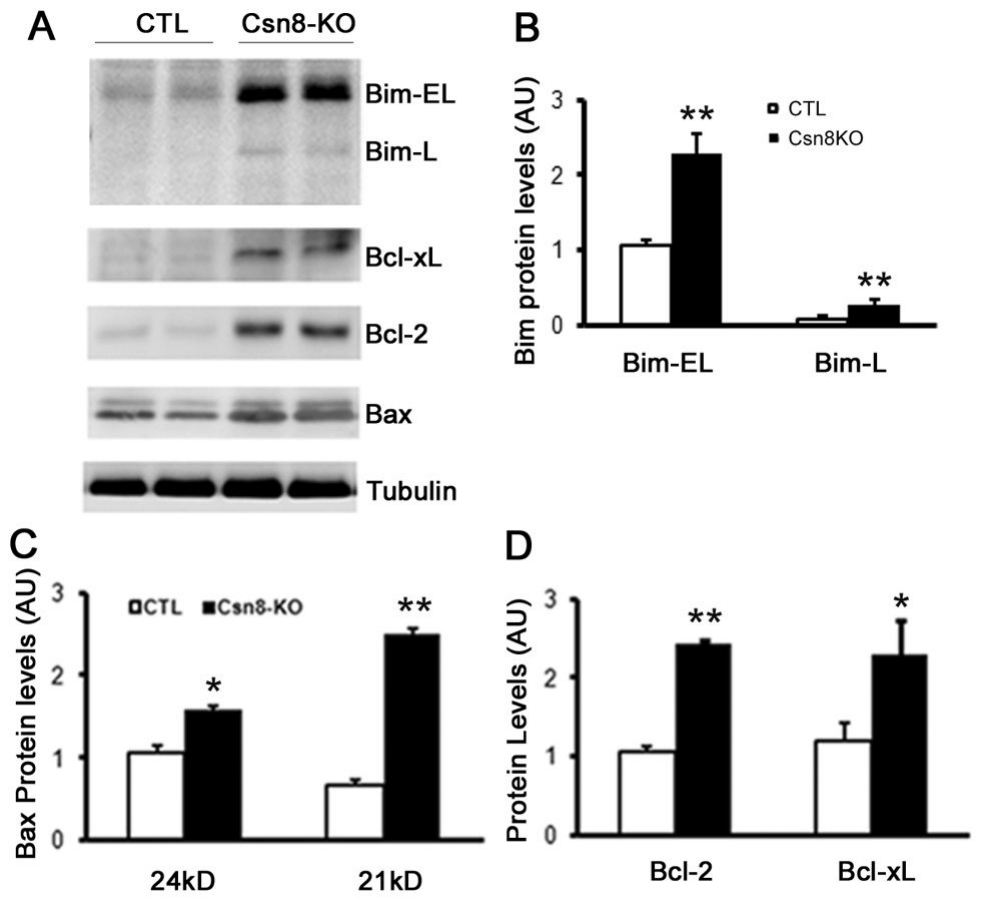
Increased protein expression of Bcl2 family members in HS-Csn8KO mouse livers. Four pairs of HS-Csn8KO and CTL littermate mice at 4-week-old were used for analyses presented here. (**A**) Representative images of western blot analyses of the indicated Bcl2 family proteins. β-Tubulin was probed as loading control (B~D). Summary of quantitative analyses. Pooled densitometry data of Bim (**B**), Bax (**C**), Bcl2 and Bcl-xL (**D**) normalized to β-tubulin are presented. *p<0.05, **p<0.01 vs. CTL; n=4 mice/group.

**Figure 8:**
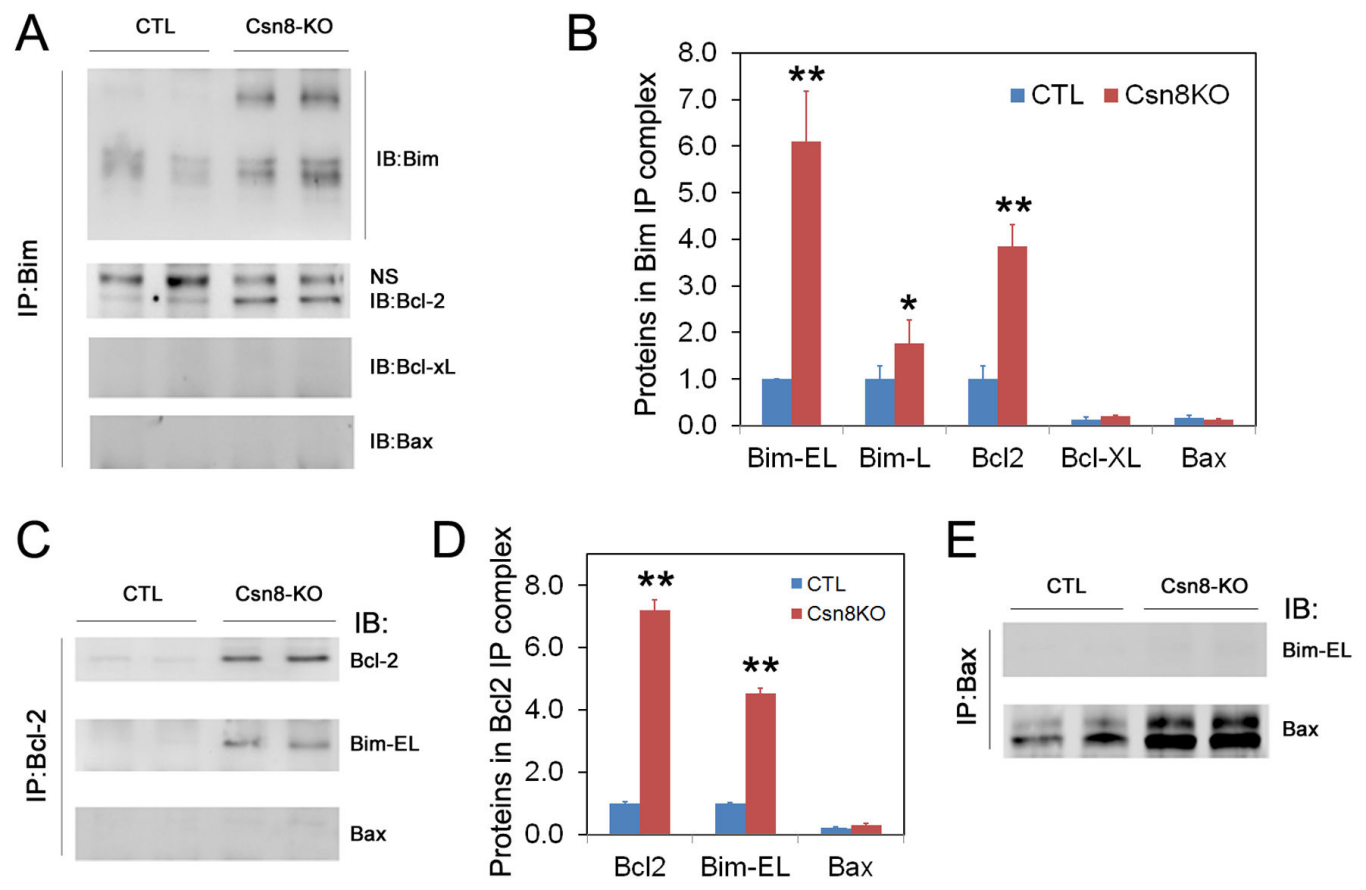
Bim forms complexes with Bcl2 but not Bax in HS-Csn8KO liver. Crude proteins extracts from liver tissues were used for immunoprecipitation (IP) followed by iimunoblotting (IB) analyses. (**A**) Immunoprecipitation using a Bim-specific antibody followed by IB for the indicated proteins. (**B**) Pooled densitometry data of the IP western blot analyses as illustrated in panel A. For each protein, the density of the Csn8KO group is shown as the relative value to the average value of the corresponding CTL group. Arbitrary unit is used. (**C**) Immunoprecipitation of Bcl2 was followed by IB for Bcl2, Bim, and Bax. NS denotes a non-specific band. (**D**) Pooled densitometry data of the IP western blot analyses as illustrated in panel C. (**E**) Immunoprecipitation of Bax was followed by IB for Bax and Bim. *p<0.05, **p<0.01 vs. CTL.

In the regulation of apoptosis, the Bcl2 family proteins play a critical role in response to specific stress signals either to promote or to inhibit cell death. Thus, we examined the expression of pro-apoptotic Bcl2 family proteins in HS-Csn8KO livers. We found that the protein levels of BH3-only protein Bim-EL and Bim-L were significantly upregulated (Figure 7A, 7B) and the multi-domain pro-apoptotic protein Bax was also expressed at a higher level compared with that in the CTL group (Figure 7A, 7C). Likewise, the protein abundance of anti-apoptotic family members Bcl2 and Bcl-XL was elevated in HS-Csn8KO livers (Figure 7A, 7D). These findings imply that disequilibrium or altered interaction between the pro-apoptotic and anti-apoptotic proteins of the Bcl2 family might be involved in Csn8 deficiency-induced hepatocyte apoptosis, prompting us to examine their interactions (see below, Figure 8).

### 6. Increased interaction between Bim-EL and *Bcl2* in HS-Csn8KO livers

Activation and polymerization of Bax and/or Bad constitute a critical step in the mitochondrial pathway of apoptosis. The mechanism by which BH3-only protein Bim activates Bax remains a matter of active debate. One possibility is the neutralization of anti-apoptotic Bcl2 family members that releases Bax from the anti-apoptotic complex and the other is for Bim to bind directly and activate Bax [23–25]. To gain insight into the mechanism of BH3-only protein Bim-induced apoptosis and its interactions with other Bcl2 family members, we performed reciprocal immunoprecipitation experiments to determine whether Bim directly or indirectly activates Bax. First, we used the Bim antibody to pull down Bim proteins from the crude protein extracts of HS-Csn8KO livers. The immunoprecipitated Bim-containing protein complexes were then probed with antibodies against Bcl2, Bcl-XL, or Bax by western blotting, respectively. As shown in Figure 8A and 8B, anti-apoptotic protein Bcl2 but not Bcl-xL was co-immunoprecipitated with Bim, indicating that Bim preferentially binds to Bcl2 in HS-Csn8KO hepatocytes. No Bax was detected in the Bim-precipitated complexes, which indicates that Bim does not bind directly to Bax in this case. Second, the antibody against Bcl2 was used to pull down the respective protein complexes, which were then immunobloted for Bim and Bax (Figure 8C and 8D). The results showed that Bim-EL not Bax was co-immunoprecipitated with Bcl2, which further confirmed that Bim most likely activates indirectly Bax via binding and engaging anti-apoptotic protein Bcl2. Lastly, we also employed Bax antibodies for immunoprecipitation. Bim was nearly undetectable in Bax-containing protein complexes from Csn8KO livers (Figure 8E). Thus, our results do not support a direct interaction between Bim and Bax, but rather suggest the exclusive binding of Bim with Bcl2 for the indirect activation of pro-apoptotic protein Bax to trigger apoptosis in HS-Csn8KO livers.

## Discussion

A growing body of evidence indicates that the CSN is involved in the regulation of a variety of cellular processes including development [37–39], cell cycle progression [40,41], DNA damage checkpoint [42], and immune responses [16,43,44]. The most studied functions of the CSN are its roles in the regulation of ubiquitination via its deneddylation activity toward CRLs. Previous studies with cardiomyocyte- or hepatocyte-restricted Csn8KO in mice have revealed that Csn8 is essential for the cell survival and the functioning of the heart and livers [17–19]. Csn8 is required for UPS function in the perinatal heart (a post-mitotic organ) but this has not been examined in a mitotic organ. In the present study, we investigated the effect of HS-Csn8KO on UPS function in livers and explored the potential molecular pathway through which hepatocyte apoptosis is activated by Csn8 deficiency. Our results reveal that the conditional targeting of the *csn8* gene in hepatocytes perturbs the UPS and leads to the impairment of UPS proteolytic function. Our data further suggest that the UPS impairment is associated with increased hepatocyte apoptosis and the latter is likely activated via a Bim-mediated pro-apoptotic signaling pathway.

### 1. Csn8 deficiency causes UPS derangement

Previous studies have demonstrated that Csn8 is required for the formation of the CSN holocomplex and the *bona fide* cullin deneddylation activity of the CSN in mammals [16,17,19]. CSN regulation of CRLs has long been believed to depend on CSN deneddylase activity but additional mechanisms independent of deneddylation were also suggested by recent reports [14,45]. Consistent with a role of the CSN in preventing self-destruction of the components of CRLs, we observed in HS-Csn8KO livers significant decreases in CIS and VHL, two SOCS (suppressor of cytokine signaling)-box-containing substrate receptors of CRLs. The CIS down-regulation was accompanied by increases of its substrate Bim-EL but decreased VHL was not associated with an increase of HIF1α, a known substrate of VHL (Figure 1A). Interestingly, some other examined substrate receptor proteins (e.g., β-TrCP, Skp2) did not show significant changes although their *bona fide* substrates (e.g., β-catenin for β+-TrCP and p27 for Skp2) were significantly increased (*data not shown*). These *in vivo* data support the notion that the CSN differentially regulates the stability and activity of CRLs.

Recent data demonstrate that not only cullins but also a number of other proteins, such as p53, epidermal growth factor (EGF) receptor, ribosomal protein L11, etc., can also be modified by Nedd8 [46]. In the present study, our examination of Nedd8 conjugates reveals a remarkable increase of HMW Nedd8-conjugated proteins in HS-Csn8KO livers (Figure 1B, 1C). These increased HMW Nedd8 conjugates are likely neddylated forms of non-cullin proteins because immunoblotting shows that they are negative for cullin1 through cullin4 that we have examined. These new findings imply that the CSN may also be responsible for deneddylation of target proteins other than cullins. It will be interesting to identify the HMW neddylated proteins accumulated in HS-Csn8KO mouse livers.

CRLs are a large family of ubiquitin ligases and are by estimate responsible for ~20% of ubiquitination in the cell [47]. It is reasonable to expect that defects in the CSN impair CRLs and thereby lead to an appreciable decrease in global ubiquitination. Similar to what was observed in the heart with cardiomyocyte-restricted Csn8KO, we detected remarkable increases in the HMW ubiquitin conjugates in HS-Csn8KO livers (Figure 1D). This suggests that the removal of ubiquitinated proteins by the 26S proteasome is likely also impaired by Csn8 deficiency. Consistent with decreased proteasomal function, the surrogate UPS substrate GFPdgn, when overexpressed in the HS-Csn8KO, was also significantly accumulated (Figure 3A ~ 3C). The marked reduction of the protein levels of Rpt5 (a critical subunit of the 19S base) in HS-Csn8KO livers (Figures 2A, 3D, 3E) may have contributed to the proteasomal impairment because Rpt5 has been elegantly shown to play an indispensable role in 26S proteasome assembly [48,49]. The mechanism for Rpt5 down-regulation is unclear and does not appear to be attributable to an increased caspase activity in HS-Csn8KO livers. Activated caspase 3 can cleave Rpt5, Rpn2, and Rpn10 of the 19S proteasome during apoptosis [50]. Indeed, hepatocyte apoptosis was immediately increased upon the depletion of Csn8 protein in the HS-Csn8KO livers and remained significantly increased during the first 6 weeks of age that were examined (Figure 6) and we have previous reported that activated (the cleaved form) caspase 3 was significantly increased in Csn8 deficient livers [19]. However, no discernible decreases of Rpn2 and Rpn10 were evidenced in the same HS-Csn8KO livers where Rpt5 decreases were observed, which stands against caspase activation as the main cause of Rpt5 down-regulation. Regardless of the cause of Rpt5 downregulation, the depletion of this key component of the19S proteasome is at least in part responsible for the impaired proteasomal function in HS-Csn8KO livers. Supporting this postulate, it has been reported that myocardial ischemic preconditioning preserves UPS function in ischemic myocardium via diminishing oxidative damage to Rpt5 [51].

The CSN has been proposed by some as an alternative and competing “lid” for the 19S proteasome [33,52]; however, as described above, none of the examined 19S lid subunits (Rpn2, Rpn8) or linker subunit (Rpn10) showed discernible changes in protein abundance upon Csn8 deficiency (Figure 2A). Interestingly, the expression of all three 11S proteasome subunits (PA28α, β, γ) was significantly increased in HS-Csn8KO livers (Figure 2C, 2D). This is likely a compensatory response to proteasome functional impairment because upregulation of 11S proteasomes by PA28α overexpression has been demonstrated to enhance proteasome-mediated degradation of misfolded proteins [4,5]. Apparently, as revealed by the increases in GFPdgn proteins, this compensatory response was inadequate. Consistent with impairment of UPS-mediated protein degradation, striking accumulation of IBL structures and increases in the nuclear and cell size of hepatocytes as shown by histological characterizations are prominent morphological features (Figures 4, 5) accompanying severe liver malfunction that was previous described for liver with HS-Csn8KO [19].

### 2. Increased Bim expression may mediate hepatocyte apoptosis by binding Bcl2 in HS-Csn8KO livers

Blocking neddylation (including cullin neddylation) by, for example, a Nedd8 activating enzyme (NAE) inhibitor (e.g., MLN4924) can effectively induce cell death in cancer cells and is being clinically tested for treating malignancy [47]. Here we show that blocking cullin deneddylation by HS-Csn8KO can also cause massive apoptosis in the liver (Figure 6). A common mediating mechanism for the two opposing approaches to cause apoptosis resides likely in the inhibition of CRLs and the resultant defects in the degradation of critical regulators of cell cycle and cell death. It is in active investigation regarding how inhibition of CRLs causes apoptosis. Inactivation of cullin neddylation by MLN4924 in cancer cells can accumulate multiple CRL substrates, including (1) cell cycle inhibitors such as p21, p27, and Wee1, resulting in cell cycle arrest [53]; (2) DNA replication licensing factors CDT1 and ORC1, leading to DNA re-replication stress and the DNA damage response [47,54]; and (3) IκBα, inhibiting NFκB activity [55]. These factors may all contribute to apoptosis induced by NAE inhibition. In the present study, we collected compelling evidence that Csn8 deficiency reduces a CRL substrate receptor protein CIS and increases its substrate Bim (Figures 1, 7). Bim is a pro-apoptotic BH3-only protein of the Bcl2 family, acting in the upstream of mitochondria-mediated apoptosis [22–24,56]. Bim has at least 18 different splicing variants, among which BimEL (extra-long), BimL (long), and BimS (short) are the three major isoforms. BimEL and BimL expression are tightly regulated at both the transcriptional and posttranslational levels [29]. In healthy cells, Bim is sequestered to the microtubule-associated dynein motor complex by binding to dynein light chain LC8, and is thereby unable to promote cell death. Upon death stimulation, Bim is released from the microtubules, promoting Bax activation and apoptosis [57,58].

In HR-Csn8KO mouse livers, both the pro-apoptotic members (Bim, Bax) and the anti-apoptotic members (Bcl2 and Bcl-XL) of the Bcl2 family were significantly increased (Figure 7), suggesting that the intrinsic apoptotic pathway has likely mediated the increased hepatocyte apoptosis induced by Csn8 deficiency. The interaction and the functional balance between pro-apoptotic and anti-apoptotic Bcl2 family proteins in a cell determine the fate of the cell [21]. Anti-apoptotic Bcl2 family members usually inhibit the pro-apoptotic activity of Bax in healthy cells. There are two competing theories for the mechanism by which the pro-apoptotic BH3-only proteins, such as Bim, promote the activation of Bax: one proposes that Bim binds to Bax and thereby activates Bax directly [26,59]; the other postulates that Bim binds and sequesters the anti-apoptotic Bcl2 family proteins, which relieves Bax from being bound by Bcl2 and allows polymerization and activation of Bax [24]. Our data favor the latter as the mechanism taken by the increased Bim to promote hepatocyte apoptosis in HS-Csn8KO mouse livers. This is because our co-immunoprecipitation reveals that increased BimEL and Bcl2 existed in the same complex, while increased Bim did not interact with Bax and the increased Bcl2 did not appear to interact with the increased Bax in HS-Csn8KO livers (Figure 8).

## Conclusions

The present study demonstrates that Csn8 deficiency compromises protein deneddylation, differentially down-regulates substrate receptor proteins of CRLs, and selectively accumulates CRL substrates such as pro-apoptotic protein Bim, and reduces 19S proteasome subunit Rpt5, leading to severe UPS functional impairment and massive apoptosis in hepatocytes of intact mice. Increased Bim expression and its interaction with Bcl2 may play an important role in mediating hepatocyte apoptosis in HS-Csn8KO mice.
